# Development of a Psychological Intervention to Promote Meaningful Activity in People Living With Mild Dementia: An Intervention Mapping Approach

**DOI:** 10.1093/geront/gnab047

**Published:** 2021-04-05

**Authors:** Vasiliki Orgeta, Eleni Palpatzis, Yi Na See, Remco Tuijt, Elisabet Sole Verdaguer, Phuong Leung

**Affiliations:** Division of Psychiatry, Faculty of Brain Sciences, University College London, UK; Division of Psychiatry, Faculty of Brain Sciences, University College London, UK; Department of Rehabilitation, National University Hospital, Singapore; Research Department of Primary Care and Population Health, University College London, UK; South London and Maudsley NHS Foundation Trust, UK; Division of Psychiatry, Faculty of Brain Sciences, University College London, UK

**Keywords:** Behavioral activation, Dementia and emotional well-being, Intervention development, Psychological theories of aging, Staying active

## Abstract

**Background and Objectives:**

Despite the importance of meaningful activity in mild dementia, only limited data are available on the development of interventions supporting people with mild dementia to engage in meaningful activity. In this article, we describe the development of an intervention that responds to this need.

**Research Design and Methods:**

Intervention mapping (IM), an evidence-based approach, was used to develop STAYING ACTIVE (STAYing well and active—schedulINg meaninGful and enjoyAble aCTIvities to promote Vitality and wEll-being in mild dementia). The first step, a needs assessment, comprised a literature review, focus groups, and individual interviews with service users. Performance objectives of the intervention were formulated in Step 2, followed by the development of theory-based methods in Step 3. In Step 4, the new intervention was developed based on data collected in previous steps, existing interventions, and pilot testing. Qualitative data were analyzed using framework analysis.

**Results:**

The needs assessment indicated that people with dementia and their carers view “staying active” as an important part of “enjoying life.” Adapting to loss through compensation and receiving support were key facilitators of engaging in meaningful activity. Ecological, psychosocial, and activity-oriented theories guided the development of theory-based intervention strategies, which were based on awareness, skills, and addressing barriers of meaningful activity.

**Discussion and Implications:**

STAYING ACTIVE is grounded on theory, and service user experiences and aims at promoting meaningful activity in mild dementia. The IM framework may be useful in the development of future psychosocial interventions for people with dementia, facilitating transparency when efficacy is evaluated.

In line with psychological theories of aging placing a strong emphasis on meaningful activity as key to “successful aging” (Martin et al., 2015), research has shown that engaging in meaningful activity increases life expectancy (Musich et al., 2018) and reduces disability in older people with and without cognitive impairment (Lu et al., 2016; Orgeta et al., 2019; Rovner et al., 2018). A considerable body of evidence shows that maintaining a high sense of purpose in life through activities contributes to living well with dementia (Hyer et al., 2008; Martyr et al., 2019; Meeks et al., 2015). Interventions that support people with dementia to sustain activities are the most effective for delaying functional decline (Laver et al., 2016) and can benefit people with dementias’ emotional well-being (Meeks et al., 2015; Orgeta et al., 2019). Interventions that promote independence and remaining engaged in activities are highly valued by people with dementia and their families (Harding et al., 2019) and are likely to be more cost-effective than premature admission to long-term care (Vandepitte et al., 2020).

Although several interventions promoting meaningful activity have been reported in the dementia literature, there are currently limited data available on intervention rationale and theoretical underpinnings (Oyebode & Parveen, 2019). In the absence of detailed information about how interventions were developed, stakeholders have limited information in terms of future planning, which can affect intervention initiation and sustainability (Walugembe et al., 2019). For example, a lack of rigorous development can result in less useful interventions, which are harder to evaluate and less likely to be implemented (Kok et al., 2016). This concern is particularly relevant for people with mild dementia and the translation of interventions into clinical practice.

Supporting people with mild dementia and their carers to sustain activities is likely to be complex, therefore empowering people with dementia and their families to maintain meaningful activity will require complex interventions, with a sound theoretical basis (Kok et al., 2016). Intervention mapping (IM) is an established approach for developing complex theory and evidence-based interventions (Bartholomew et al., 2016), reflecting the complexity of decision-making processes of designing interventions which are collaborative, iterative, and cumulative (Kok et al., 2016).

This article describes the development of a new intervention using IM, STAYING ACTIVE (STAYing well and active—schedulINg meaninGful and enjoyAble aCTIvities to promote Vitality and wEll-being in mild dementia), which was aimed at supporting people with mild dementia to optimize and maintain meaningful activity. Despite the term *meaningful activity* being commonly used in the aging and dementia literature, there is currently no consensus on its definition (Phinney et al., 2007). For the purposes of this study, we defined meaningful activity as any activity that was related to a person’s interests and roles, involved active participation, and supported individuals’ need for identity and belonging (Eakman, 2012; Genoe & Dupuis, 2014).

In this work, we describe the different processes of the STAYING ACTIVE intervention development in detail, by presenting data on the first four steps of IM. These were (a) a needs assessment, (b) identification of performance and change objectives of the intervention, (c) selection of theory-based intervention methods and practical applications, and (d) development of the new intervention. Our secondary aim was to provide an example of how IM can be applied to develop complex community-based interventions for people with mild dementia and highlight the potential benefits of this approach.

## Method

**Figure 1.**
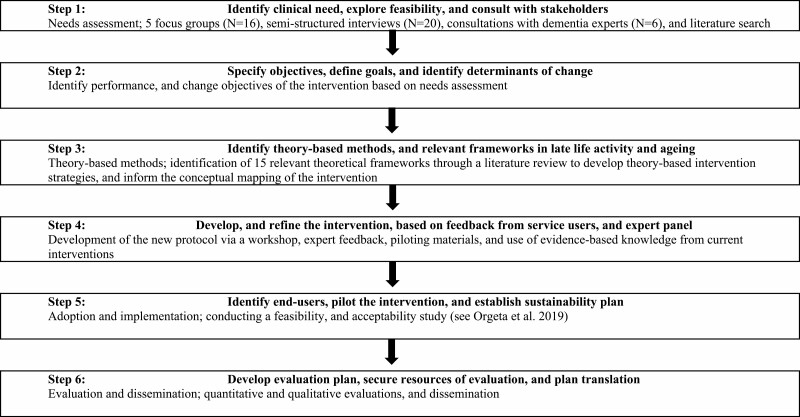
Adapted Intervention Mapping used to develop the STAYing well and active—schedulINg meaninGful and enjoyAble aCTIvities to promote Vitality and wEll-being in mild dementia (STAYING ACTIVE) intervention.

The different stages of IM used to develop STAYING ACTIVE are described in Figure 1, in a linear fashion; however, as is often the case, there was frequent movement between the steps and when iterative changes were required. Step 1 describes our needs assessment of meaningful activity in mild dementia, followed by Step 2 which presents how performance and change objectives of the intervention were derived. Step 3 reports on the process of developing theory-based methods and Step 4 on the development of the intervention protocol and materials. A successive article presents results of program implementation and evaluation (Steps 5 and 6 of IM), supporting the feasibility and acceptability of STAYING ACTIVE in a U.K. setting (Orgeta et al., 2019), and will therefore not be presented in this article.

Prior to developing the intervention, we formed a consultation team of experts, which included an old age psychiatrist, a clinical psychologist working with older people, two dementia care professionals with backgrounds in social care, and four family carers. We worked closely with this group across four qualitative roundtable meetings, one for each step of IM. We used their feedback to inform our needs assessment (Step 1), codevelop performance objectives of the intervention (Step 2), and identify relevant theories to inform theory-based intervention strategies (Step 3). Expert knowledge was then used to codevelop and refine intervention materials for Step 4 and a conceptual model of STAYING ACTIVE to inform a full-scale clinical effectiveness trial.

### Step 1: Needs Assessment of Meaningful Activity in Mild Dementia

The purpose of Step 1 was to assess the current need for meaningful activity in mild dementia by identifying both behavioral and environmental determinants, combining both qualitative and quantitative methods (Bartholomew et al., 2016). Our needs assessment commenced with a systematic review of activity-based interventions for older people, which aimed toward identifying effective interventions, their performance objectives, and outcomes targeted (Orgeta et al., 2017).

We then focused on a problem analysis of meaningful activity in mild dementia, by incorporating experiences by service users. We conducted a series of focus groups and semistructured individual interviews with people with mild dementia and carers to explore perceptions of “staying active” and identify barriers and facilitators of engagement in meaningful activity. We recruited people with (a) mild dementia of any type living in the community, through National Health Service memory clinics in London, UK, and (b) family carers supporting people with mild dementia living in community settings. Our focus groups and individual interviews followed a specific topic guide (Supplementary Table 1) codeveloped with our expert group, which was refined as the study progressed. We asked what people with dementia and carers felt “staying active” meant, and what they considered to be the most important obstacles to staying active. We additionally explored which activities were considered as meaningful and what helped maintain activities in everyday life. All participants gave informed consent prior to the interviews. The London—Camberwell St Giles Research Ethics Committee approved the study (REC 16/LO/0540).

**Figure 2.**
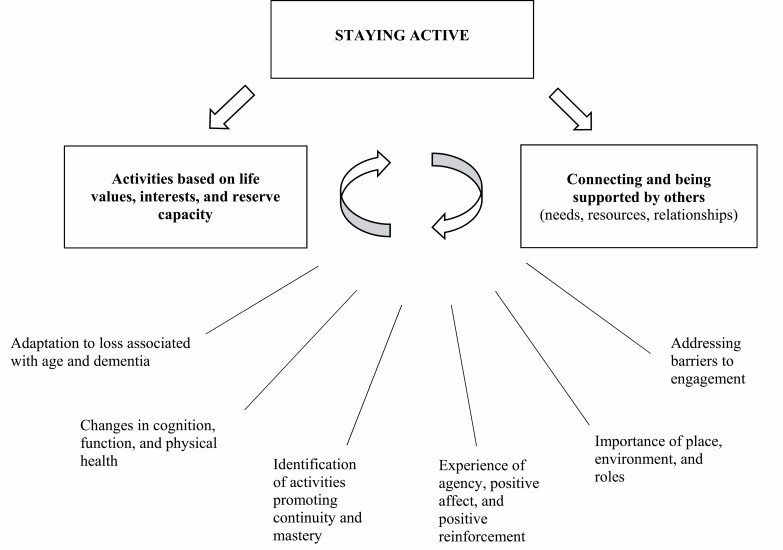
Conceptual model of STAYING ACTIVE.

We used the method of triangulation (Farmer et al., 2006) to integrate findings of the systematic review, focus groups, and individual interviews. After identifying themes from each data source and sorting these into similar categories, we “convergence coded” data to identify areas of agreement and disagreement. We then discussed results with the expert group and refined our coding based on these discussions. We applied this method across all steps of IM and summarized data into our preliminary problem-focused model (Figure 2). This model outlined the key determinants upon which the intervention needed to focus, identifying both individual and contextual factors of meaningful activity, which was refined across all IM stages.

### Step 2: Performance Objectives and Change Objectives of the Intervention

The purpose of Step 2 was to identify what should change as a result of the intervention (Kok et al., 2016). We used data of the systematic review (Orgeta et al., 2017), focus groups, individual interviews, and expert feedback (Step 1) to inform the performance objectives of the intervention and the factors influencing whether or not these are carried out, highlighting that Steps 1 and 2 were iterative. To create performance objectives, we asked (a) What are the essential components of the intervention? and (b) What people with dementia and carers will need to do to engage and maintain meaningful activity? Relevant and changeable determinants of meaningful activity were then plotted on a matrix of performance and change objectives. These were then presented in a roundtable meeting to our expert group and refined via a triangulation approach as described above.

### Step 3: Developing Theory-Based Methods

Step 3 involved identifying theoretical methods that contribute to achieving change objectives and practical applications that help operationalize these methods. Therefore, in this step, developers provide clear information on how the intervention may influence behavior. This step consisted of developing a matrix of theoretical determinants of meaningful activity in older people and theory-based strategies matched to each performance objective of the intervention. We identified both individual and environmental theory-based methods in line with IM taxonomy (Kok et al., 2016).

We used theory-based methods that were both general and specific to the context of mild dementia. General theory-based methods were awareness, attitudes, and skills, and environmental methods were carer support, social support, and community involvement. For dementia-specific theory-based methods, we reviewed theories of aging to identify individual and environmental parameters influencing activity engagement that were theory-driven. We identified relevant theories by searching four online databases (Medline Ovid, Embase, PsycINFO, and CINAHL) using a comprehensive search strategy, comprising terms such as “meaningful activity,” “activity theory,” “older people,” “dementia,” “theory,” and appropriate synonyms (Supplementary Material A).

In the next stage, practical strategies were identified and incorporated into the intervention design, specifying which practical strategy preserved parameters for effectiveness for each session (Kok et al., 2016). For example, a parameter for identifying activities was that people with dementia and carers worked together with the therapist to identify activities and barriers, as opposed to the therapist simply providing instructions.

### Step 4: Development of the Intervention Protocol and Materials

The next step was to develop the intervention protocol and materials. As part of this step, we designed the intervention workbooks for people with dementia and carers, drafted the content, pretested, and further refined these to produce the final materials. To achieve this, we completed a series of activities. First, using transcripts from the focus groups and individual interviews that were independently coded by two researchers using inductive coding, we developed documents for each session. We used visual guides as much as possible based on feedback that “having pictures is better than words,” and comments from focus groups and individual interviews (Step 1) about content, and specific activities to be incorporated. A program manual was then developed that included all intervention sessions.

We then piloted the manual with 15 dyads of family carers and people with dementia who took part in Step 1, to collect input on engagement with the materials, timing, and suitability of content. The program manual also received input via a 1-day workshop with a team of psychology graduates delivering psychosocial interventions in dementia, dementia care professionals, and family carers. The purpose of this workshop was to gain input on issues around engaging with families as identified by the pilot phase. The program manual was then refined based on feedback by the piloting and workshop.

Third, using the information collected above, we embedded further practical methods into the intervention such as the use of a diary and a workbook with examples of activities (Orgeta et al., 2017; Polenick & Flora, 2013). For instance, at the start of each session, therapists were encouraged to engage in informal discussions about enjoying life and identify activities people with dementia currently engage in. This activity reinforced collaboration between therapists, people with dementia, and carers in identifying activities. After incorporating data from Steps 1–4, another round of refinement took place, before the final version of the manual was adopted.

### Data Analysis

Focus groups and individual interviews were audio-recorded and professionally transcribed. Data from the expert roundtable meetings were transcribed by the first and last authors. All qualitative data were independently analyzed by two researchers using framework analysis (Ritchie & Lewis, 2003). This involved (a) familiarization with each interview and considering important contextual notes, (b) coding, by applying a paraphrase used to classify the data, (c) developing a working analytical framework by grouping codes into categories, (d) applying the analytical framework by indexing transcripts using the existing codes, and (e) charting by summarizing data in categories derived from each transcript. We used inductive coding to ensure important aspects of the data were not missed and although we followed a specific discussion guide, we coded both substantive behaviors in addition to emotion, values, and methodological elements derived by the data (Gale et al., 2013).

For the systematic review of theories, two researchers worked independently to identify relevant theories, extract data on the main premise of each theory, and proposed mechanisms. We used thematic theoretical analysis to categorize theories (Boyatzis, 1998). Inclusion criteria for a theory to be relevant were (a) being related to activity in late life or dementia, (b) addressing facilitators or barriers to activity engagement, and (c) concerned with the emotional well-being of older people with or without cognitive impairment. We first narratively summarized each theory by describing key concepts, and in a separate meeting with our expert group, we identified key assumptions, commonalities, and differences, drawing out implications for activity engagement in people with mild dementia. Informed by these summaries, we mapped how age-related, environmental, and psychosocial factors may influence activity engagement in people with mild dementia (Figure 2).

## Results

### Step 1: Needs Assessment of Meaningful Activity in Mild Dementia

We conducted five focus groups: two with people with dementia, two with family carers, and a mixed group with both people with dementia and carers (*n* = 16). We also individually interviewed 10 caregiving dyads (*n* = 20). For demographic characteristics of the sample, see Supplementary Table 2.

*Qualitative analyses of focus groups and individual interviews about perceptions of staying active in mild dementia by people with dementia and family carers*.

Framework analyses resulted in the identification of four independent themes in relation to perceptions of staying active in mild dementia, which were *enjoying life*, *staying active*, *facilitators*, and *barriers* (Table 1).

**Table 1.** Qualitative Themes and Example Quotes of Staying Active in Mild Dementia

Themes and subthemes	Example quotes
*Enjoying life*	
Acceptance of loss	You have to accept it, you can’t do many things any more, and some things you do less well.
Being as active as you can	I have a lot more problems now but I enjoy life, I can do things I like and enjoy, it’s getting active when you can.
Being in good health	I have my health so I can enjoy life.
Feelings of happiness	You can be happy, and enjoy life no matter your medical condition.
Value the “place”	It’s really about having the right people around you. He enjoys being in a nice place, being around nice people.
*Importance of staying active*	
Awareness of staying active	I want to keep busy, I like doing things. I do a lot around the house, doing things and thinking about it makes me realize I do quite a lot.
Connecting with others	Talking to other people, it stimulates you, keeps you going. She interacts with people, she is happy.
Emotional well-being, self-esteem, and protection from low mood and worry	Activities keep spirits up. It’s about improving her confidence, doing things. Keeping her busy, changes the focus from the worry.
Continuity of roles, and the self before and after dementia	I was very active before, you can’t just stop.
Supporting activities helps carer coping	I often arrange things he can do outside the house, so he can be active a few days a week.
*Facilitators of staying active*	
Compensation via the use of strategies and problem solving	You really need to find shortcuts, do things that are easy, take less time and energy. We are a good team together, we talk things through.
Resources (accessing support by others, physical resources, health)	My son takes me out, I walk, its good for me. Having a car is very important. Having good health, and looking after yourself and your diet helps.
Activities provide meaning	Activities give you a sense of purpose, something to do, but it has to mean something.
Place and living in a positive environment	My husband wants to do everything, so I just keep doing things with him, it motivates me. It’s being around people who understand what dementia is.
Caregivers encouraging activity	She will always say no at first, but then she will say, that was good wasn’t it?, you just have to keep trying.
*Barriers to staying active*	
Physical health, chronic illness, accidents, and loss of mobility	I like to go out and about but I am too slow, I just can’t keep up. You do get tired, and it’s not just the dementia.
Loss of social contact	You lose people, you don’t see others that much. Lots of gates are closed when you have dementia, you can watch, but can’t take part. Living alone can make it hard to stay active.
Memory loss and feelings of frustration and worry	If she can’t get something to work, if she forgets she does get frustrated. I worry about what would happen to her.
Carer burden on physical and mental health	I was unwell, it was all too much, I was in a bad place. I do sometimes become angry at her, I get frustrated, I realise that.

#### Theme 1. Enjoying life

Overall, participants endorsed that engaging in activities allows you to “enjoy life” and that despite challenges people can still live well with dementia. Two key concepts identified were *accepting loss* and *valuing the place*. Participants talked about accepting and adapting to loss in the context of memory decline, which facilitated living well with dementia despite this being described by most participants as a difficult process. Valuing your current living situation was important in maintaining a positive outlook such as living in a town you enjoy and being surrounded by family, relatives, and friends:

You have to accept it, you can’t do many things anymore, and some things you do less well, when you lose something you’ve been doing for years, it’s tough, … but I can still do some things, I can enjoy life. Person with dementia—Participant 1

Another participant said,

I have always lived here, I like my town, and I am happy at home, I love my surroundings very much. Person with dementia—Participant 4

Participants discussed how living with chronic illness and dementia affects the ability to live well. In fact, most participants defined enjoying life as being synonymous with *being in good health*, with living well being directly linked to individuals’ physical health. Being in good health served as an important condition to remain independent, which similarly to memory loss required adapting to physical changes taking place as a result of age.

I have my health so I can enjoy life … illness is very common, you have to learn how to adjust, … health is a big thing …, it helps you stay active and live well. Person with dementia—Participant 5

Participants talked about *being as active as you can* and how activities allowed one to experience *feelings of happiness* and *enjoying life as much as you can*. Almost all participants described “enjoying life” as an opportunity to experience *positive emotion* in everyday life and that despite “living with loss” individuals can still achieve happiness.

I have a lot more problems now but I can still do things I like and enjoy, it’s really getting active when you can when you have dementia, I think you can be happy and enjoy life no matter your medical condition ... I was very active before, I want to maintain that as much as I can. Person with dementia—Participant 4

#### Theme 2. The importance of “staying active”

Both people with dementia and carers acknowledged that staying active was an important part of everyday life and that *being aware of how active you are* promotes general well-being and remaining independent. Staying active was often perceived as protecting individuals’ *well-being* by reducing feelings of worry and low mood and was often seen as an important source of maintaining *self-esteem*. Remaining engaged in activities was important in the context of functional decline and was often related to fear of future deterioration due to dementia. Engaging in activities was seen as a way of connecting to “life before and after diagnosis,” by allowing people with dementia to *continue their roles*, habits, and preferences. Engaging in activities provided opportunities for people with dementia to express themselves and exert control over their immediate environment, via experiencing competency, despite “quietening down,” which reflected the impact of dementia on everyday activity levels.

I want to try and keep independent, and just be aware of things I can still do, going on as normal if I can, I am aware I have to keep the body active, I was very active before, you can’t just stop. Person with dementia—Participant 3

One carer mentioned:

It’s about improving confidence, doing things … she likes to be in control of things in her own house, memory loss can take that away … keeping her busy, changes the focus from the worry. Family carer—Participant 2

Engaging with others through activities was seen as an important source of motivation to “keep going,” exchange ideas, and the need to be mentally and physically stimulated. Many participants talked about how being stimulated and just “being one of the crowd” was something that was not very common after receiving a diagnosis of dementia.

Activities keep spirits up … I want to keep busy, … and talking to other people, it stimulates you, gives you things to do, you can’t be on your own, I do social gatherings, I like being with others. Person with dementia—Participant 7

Staying active was described as an important *coping strategy for carers* with many participants describing how they promoted and organized activities on a regular basis which formed part of their “routine.” Carers stressed the importance of being active outside of the home, which motivated people with dementia, and supported their physical well-being.

I often arrange things he can do outside the house, so he can be active a few days a week, … it’s a matter of routine, … I do try to introduce things, they need the mental stimulus to keep going with the routine and being physically active. Family carer—Participant 6

#### Theme 3. Facilitators of staying active

*Compensation* was described as an important facilitator of staying active, which was successful via accessing *resources* and deriving *meaning* through activities. Participants talked about how using specific techniques and problem solving allowed them to adapt to changes associated with a decline in memory and function. People with dementia perceived deriving meaning from activities as an important motivator of staying active. Receiving support directly from others, staying in good health, and looking after yourself enabled engagement with activities, with practical facilitators being accessing transport.

You really need to find shortcuts, do things that are easy, take less time and energy ... Activities give you a sense of purpose, something to do, but it has to mean something. Person with dementia—Participant 1

My son takes me out, I walk, it’s good for me, having good health, and looking after yourself and your diet helps ... Having a car is very important, so you have a way of getting to things. Person with dementia—Participant 4

Both people with dementia and carers discussed the importance of *place*, to describe how positive environments facilitated engagement in activity. Living in a supportive environment where people around you understand the symptoms of dementia and the progression of the disease was seen by many as enabling people with dementia to stay active. The value of the place was strongly related to *continuous engagement* by carers, with most participants viewing carers’ input and support in initiating activities as essential in supporting and maintaining activities over time.

It’s being around people who understand what dementia is … being with others is a good stimulus, it can get people interacting, its very stimulating for him to see friends and socialise. Family carer—Participant 6

#### Theme 4. Barriers to staying active

Declining *physical health* was the most common barrier to activity engagement, followed by *losing social connections*, and *symptoms of dementia*. Health-related barriers were consistently described as having the greatest impact on lack of engagement in activities. Declining physical health as a result of age, living with chronic illness, or experiencing an accident or a fall were described as hindering activities. Many people with dementia reported experiencing a decline in their everyday mobility, which was often associated with lack of energy and experiencing high levels of fatigue which directly influenced activity levels.

I like to go out and about but I am too slow, I just can’t keep up … You do get tired, and it’s not just the dementia. Person with dementia—Participant 1

People with dementia mentioned that loss of *social connectedness* or living alone increased the risk of not being active.

You lose people, you don’t see others that much … lots of gates are closed when you have dementia, you can watch, but can’t take part. Person with dementia—Participant 7

The most common barriers directly related to dementia were experiencing memory loss, which was often a source of worry and frustration, for both people with dementia and carers.

If she can’t get something to work, if she forgets she does get frustrated … The worry stays until the problem is solved and can be lots of things. Family carer—Participant 2

Carers experiencing high levels of *caregiver strain* due to the demanding role of being a carer hindered activity engagement. Strain due to caregiving was affecting carers’ physical and mental health and was often described as posing a negative impact on the caregiving relationship, limiting carers’ vitality and holding them back from their own life.

I was unwell, it was all too much, I was in a bad place … Sometimes it’s like being held back, from things you want to do, I want him also to do things that do not involve me; I have my own network and things. Family carer—Participant 6

Qualitative data collected in Step 1 informed intervention development by adopting a positive focus on “enjoying life” by incorporating activities that people with dementia already engage in. Adding activities that were person-specific, a source of pleasure, and those that allowed exercising mastery were identified as important strategies of the intervention. Themes on facilitators of and barriers to staying active resulted in adding a session around accessing support by others and reinforcing mobilization of resources available, thus promoting social connectedness (immediate family, friends, and wider community). Additional intervention components included adding specific information around the changing needs of people with dementia (i.e., memory loss affecting initiating and remembering activities).

### Step 2: Performance and Change Objectives of the Intervention

The goal of this stage was to define the specific intervention goals and determinants of the intervention. We used the results of Step 1 to inform the performance and change objectives of the intervention. These were then discussed via a consultation meeting with our expert group and by reviewing data collected in Step 1. After identifying the intervention objectives, the expert group examined which of these would be most suitable and easier to maintain for people with mild dementia and refined these accordingly.

Performance objectives were designed to be session specific and action-oriented. These were (a) introduction to behavioral activation principles and gathering participants’ views of engagement in meaningful and enjoyable activities (MEAs); (b) identifying MEAs and discussing their importance to everyday life; (c) identify important life areas for the individual; (d) discuss existing MEAs, identify new ones, and discuss barriers by developing a plan to address them; (e) learning relaxation techniques; (f) review of activities and discussion of facilitators and barriers for each activity; (g) developing a plan for the future; and (h) reviewing successes of the program and how activities can be maintained over time. Details of the performance objectives of the intervention are presented in Table 2.

**Table 2.** Performance Objectives of the STAYING ACTIVE Intervention

	Determinants				
Performance objectives	Attitude and belief	Knowledge and awareness	Autonomy and continuity of roles	Mastery and competence	Support by others
Dyads are introduced to the concept of BA and wish to engage in MEAs	Feel positive about an active life	Aware of the benefit of activities, deriving a biopsychosocial history	Express the importance of maintaining autonomy and continuity of roles in line with life values and interests	Activities are a source of mastery and are aligned to personal interests	Feel supported by the therapist to engage in MEAs
Dyads identify MEAs and discuss their importance for everyday life	Feel positive about the current level of activities	Aware of the benefit of engagement in MEAs	Feel confident about current activities	Gaining mastery is an important part of activities	Areas of support across several life areas are identified
Dyads discuss important life areas (leisure, social, hobbies, family and friends, health, political and spiritual interests)	Feel positive about making MEAs plans in line with life values	Discuss things that get in the way of engaging in MEAs	Feel confident about activities being in line with life values and interests	At least one area of mastery is identified and maintained throughout the intervention	Specific sources of support are identified and how these can act as facilitators
Dyads work collaboratively with the therapist to identify new or existing MEAs and address barriers to engagement	Recognize challenges because of dementia and identify barriers and facilitators	Discuss ways to overcome barriers and the value of trying	Feel confident in trying new activities and revisiting old interests	Activities that are a source of mastery are continuously reviewed	Family and significant others are mobilized to support MEAs
Dyads learn with the support of the therapist relaxation techniques	Awareness of the need to engage in relaxation and stress reduction activities	Aware of the value of relaxation activities and techniques in everyday life	Feel confident and supported in engaging in relaxation exercises	Opportunity to master a relaxation technique	Support is identified in practicing relaxation exercises and engaging in MEAs that promote relaxation
Dyads review activities and discuss potential facilitators and barriers for the future	Feel positive about addressing future challenges	Aware of barriers and how these can be overcome	Feel confident about overcoming barriers and problem solving	Discuss how mastery contributes to an active everyday life	Areas of support that are key for the future are identified
Dyads develop a plan of staying active and well for the future	Discuss a plan for maintaining MEAs	Aware of the value of continuous engagement in MEAs	Feel confident MEAs can be maintained in the future	Discuss how mastery can be maintained in the future	A plan is developed on how support can be maintained
Dyads review successes of the intervention and discuss the plan of staying active and well for the future	Review of successes and discussion of the plan for maintaining MEAs	Aware of achievements and the value of continuous engagement in MEAs	Feel confident about what has been achieved and which MEAs can be maintained in the future	Discuss what helps maintain mastery and how this can be maintained in the future	The plan of how support can be maintained is reviewed

*Note:* STAYING ACTIVE = STAYing well and active—schedulINg meaninGful and enjoyAble aCTIvities to promote Vitality and wEll-being in mild dementia; BA = behavioral activation; MEAs = meaningful and enjoyable activities.

### Step 3: Developing Theory-Based Methods

Our search of relevant theories resulted in a total of 2,626 records, of which 40 were screened for eligibility. Of these, 15 were identified as directly relevant to our study, which after discussions with our expert group were categorized as *activity-orientated*, *psychosocial*, and *ecological*. Main themes and proposed mechanisms for each theory are described in Supplementary Table 3.

#### Activity-orientated theories

We identified three theories making specific hypotheses about how older people maintain positive functioning through activities. The selective optimization with compensation (SOC) framework (Baltes & Baltes, 1990), the successful aging paradigm (Rowe & Kahn, 1998), and the activity theory of aging (Cavan et al., 1949) were considered relevant to our intervention due to their emphasis on *active engagement with life* and its relationship to well-being. Common themes included *adaptation to loss* through *activities*, which provide *meaning*, and are linked to a person’s *life story.* In line with activity-oriented theories access *to resources* supports *active engagement* with life, with *autonomy* and *mastery* being important related constructs.

#### Psychosocial theories

We identified several psychosocial theories making specific hypotheses about how activities maintain well-being in late life allowing older people to adapt to loss associated with age. Continuity theory (Atchley, 1989) argues that *habits* maintained over an individual’s life span support older people’s attempts to cope with loss, through c*ontinuity of interests* and *roles*. Self-determination theory (Deci & Ryan, 1985) argues for *intrinsic motivation*, whereby experiencing competence, and meaningful social connections are fundamental human needs. According to Heckhausen and Schulz’s life-span theory of control (Heckhausen & Schulz, 1995), individuals exert *primary control* over their environment, in order to enhance their well-being (Atchley, 1991), whereas identity theory argues that active participation in life allows for continuous *identity formation* (Erikson et al., 1986). In Bandura’s social cognitive theory, individuals derive their *sense of self* by experiencing *personal agency* (Bandura, 1986), whereas socioemotional selectivity theory (Carstensen, 1992) places time constraints at the center of older peoples’ focus on *emotion-oriented goals* driven by activities that *maximize positive affect*. The copying and adaptation with active aging model (Salazar-Barajas et al., 2017) argues that *active aging* represents an adaptation strategy to *disease-related disability*, whereas the ways of coping model (Tanner, 2007) describes *valued activities* as an important contributor of *a sense of* “doing well.” Two theories identified specific to dementia were the hierarchical model of needs (Scholzel-Dorenbos et al., 2010) and the stress process model in early dementia (Hilgeman et al., 2014). These theories posit that people with dementia are motivated toward *meaning-based coping*, whereby *acceptance of loss* and *feelings of continuity* support *autonomy* and *adaptation to loss*.

#### Ecological theories of aging

Three ecological theories were identified as directly relevant to meaningful activity in mild dementia, viewing active aging as an interplay between individuals’ function and his/her adaptation to physical and social changes associated with age. These were the ecological model of aging (Lawton, 1980), the ecological framework of place (Moore, 2014), and the preventive corrective proactive model (Kahana et al., 2014). These models place an emphasis on *agency*, conceptualizing behavior as a function of the *competence* of the individual, and their connection with their environment, with the *place* being central in maintaining meaning, derived through *roles*, and maintaining *autonomy* which supports coping with *environmental constraints*.

Identification of relevant theories informed STAYING ACTIVE by understanding the context of activity engagement in mild dementia and the importance of addressing barriers at individual, interpersonal, and social levels. As a result of these analyses, we incorporated intervention strategies that enabled engagement in activities that were person-centered in line with the person’s values and interests, thus promoting continuity, and those supporting mastery and agency. Details of how the intervention was informed by the different stages of IM are presented in Supplementary Table 4.

### Step 4: Development of the Intervention Protocol and Materials

At Step 4, our aim was to develop the intervention protocol and associated materials. We initially drafted a manual adapted by existing interventions, informed by the systematic review of activity-based interventions for older people (Orgeta et al., 2017). We then incorporated data from the focus groups and individual interviews and results of Steps 2 and 3 and presented our draft manual in a roundtable meeting to our expert group. Key issues identified were ease of presentation, flexibility, and using a mode of delivery that allowed for a person-centered approach. The manual was further refined to accommodate these comments.

Second, we incorporated data of the workshop with family carers, clinicians, and graduate psychologists experienced in delivering psychosocial interventions. Refinements at this stage included simplifying some of the intervention materials, adding specific examples of activities, and developing training manuals by adopting specific intervention scenarios. Third, we created eight individual manualized sessions (each 60–90 min in duration), which we piloted with 15 dyads to test the readability of materials. This form of testing aimed toward assessing whether the program materials and messages were relevant, culturally acceptable, and comprehensible by the target population. The purpose of this step was also to identify any potential problems with implementation.

Fourth, we presented the session materials to our expert group and refined sessions by considering feedback from our piloting. Changes at this stage included organizing sessions thematically depending on life areas that were important for each individual. Additional adaptations included incorporating a biopsychosocial history throughout the different sessions to ensure the intervention is adapted at the individual level. This was facilitated at a practical level by introducing specific life areas to be addressed by the intervention such as “leisure and social activities,” “looking after my physical health,” “family and friends,” “political and spiritual interests,” “voluntary work,” and “accessing resources and support.” Practicing relaxation and physical activity exercises were considered very important by both family carers and people with dementia and were included in the sessions. Both, however, were adapted and simplified to reduce the cognitive load for participants. Feedback from piloting at this stage indicated that eight sessions were appropriate to achieve the intervention objectives.

The final intervention components included increasing pleasant events and rewarding activities daily, practicing relaxation exercises aimed at stress reduction, and promoting both physical and social activities. A detailed description of intervention components of STAYING ACTIVE is presented in Supplementary Table 5.

## Discussion and Implications

In the present study, we used IM to develop the first theory-based intervention promoting meaningful activity in people with mild dementia. Adopting the IM framework was proven useful in ensuring that the development of the intervention was comprehensive, systematic, and thorough, with a sound theoretical basis. IM may therefore be useful in the development of complex psychosocial interventions for people with dementia, facilitating translation of interventions into clinical practice, and allowing for greater transparency when intervention efficacy is investigated (Kok et al., 2016).

Results of our qualitative analyses, which formed part of our needs assessment, showed that engaging in meaningful activity allows people with dementia to buffer the negative effects of losses associated with dementia. These findings are in line with theories of “successful aging” and coping with age-related adversity (Cavan et al., 1949; Rowe & Kahn, 1998), providing further evidence for the clinical need of these types of interventions. A key finding of our qualitative data was that activities support people with dementia to adapt to loss via *active aging* (Cavan et al., 1949), fostering a positive sense of self (Atchley, 1989; Phinney et al., 2007).

Consistent with SOC theory, participants described a process of adaptation that was characterized by the use of *compensation*, such as selecting activities that are meaningful, those that allowed them to exercise mastery, and accessing *resources*, such as relationships with significant others (Cavan et al., 1949). An important contribution of our study is the finding that despite the significant narrowing of activities, people with dementia discussed about the need and importance of maintaining high levels of function in several life areas. This finding was observed in both participant and carer data, with areas of mastery maintained being directly influenced by the person’s life trajectory, and reserve capacity (Atchley, 1989; Phinney et al., 2007).

A common mechanism identified across several theories and our qualitative data was experiencing *agency*, where activities function as catalysts, supporting people with dementia to age in *place* (Baltes & Baltes, 1990; Lawton, 1980). In line with both theory and empirical work, mastery emerged as an important psychological resource throughout all stages of intervention development, facilitating adaptation to loss (Genoe & Dupuis, 2014; Rowe & Kahn, 1998). This form of *continuous engagement* may be particularly important for people with dementia, which due to environmental and motivational barriers as opposed to lack of capacity are more likely to discontinue involvement in meaningful activity (Harding et al., 2019). In line with several theories, activity participation in the context of mild dementia may support maintaining mastery and deriving a sense of meaning, which in turn may contribute to increasing and maintaining well-being (Kahana et al., 2014). This finding is supported by preliminary results that interventions promoting meaningful activity may benefit people with dementia and mild cognitive impairment in a variety of settings (Meeks et al., 2015; Orgeta et al., 2019; Rovner et al., 2018).

### Implications for Care and Future Research

We found that most people with dementia discussed the ability and need to maintain high levels of activity in several life areas (maintenance of household, engaging with their local communities, maintaining hobbies); this reflects an important adaptation strategy, emphasizing the important role that motivational factors and resources may have in supporting people with dementia and their families lead “active lives” (Harding et al., 2019). The importance of “place” is consistent with recent recommendations that maintaining high levels of activity and remaining socially integrated is an important psychosocial need for people with dementia and should be a core outcome for future community-based interventions (Harding et al., 2019; Meeks et al., 2008).

Understanding barriers to activity participation is important for the development of interventions aimed at promoting meaningful activity in people with dementia. Interestingly, many of the barriers identified across the different stages of intervention development were directly related to age and disease as opposed to dementia per se. Age-related changes in mobility, accidents, chronic illness, and decline in physical health were among the most significant barriers identified by both people with dementia and family carers. This observation may be particularly important for people with mild dementia and suggests a need for interventions that promote both physical and emotional well-being.

### Limitations

Despite the originality of our findings, this study has several limitations. Although we reviewed several theoretical models to identify those most relevant, we may have still missed important theories. Most people with dementia taking part in our study were generally very active and did not experience significant comorbidity. Most of the sample consisted of people who had regular contact with their local memory clinic. We were unable to recruit people with no regular carer or people experiencing significant communication difficulties. Lastly, although we did include people with dementia at Steps 1 and 4 of intervention development, our expert group did not include people living with dementia, and we did not consult occupational therapists when developing the intervention.

## Conclusions

This study describes the development of STAYING ACTIVE (Orgeta et al., 2019), as a theory-based and user-informed community-based psychological intervention aimed at promoting meaningful activity in people with mild dementia. The STAYING ACTIVE program has been enhanced by the use of IM, which advocated for attention to detail in all aspects of the development process, including content derived by theory, and service user perspectives and experiences. Our study contributes important new knowledge to current theories and future care models for people affected by dementia. Promoting meaningful activity in people with dementia is likely to be key in future service care provision in dementia, as conceptual and empirical work in this area develops.

## Funding

This project was supported by a grant from the Alzheimer’s Society 259 (AS-SF-15-005 to V. Orgeta).

## Conflict of Interest

V. Orgeta and P. Leung are authors of a Cochrane review on psychological treatments for people with dementia. V. Orgeta received a personal senior fellowship award to undertake this work. There are no other known conflicts of interest. All other coauthors report no conflict of interest.

## Supplementary Material

gnab047_suppl_Supplementary_Materials

## Data Availability

The full data set can be requested from the corresponding author. The full protocol can be accessed here: https://bmjopen.bmj.com/content/8/2/e021074.
