# Supplementary material for: Development of a Psychological Intervention to Promote Meaningful Activity in People Living With Mild Dementia: An Intervention Mapping Approach
Source: Gerontologist. 2021 Apr 5;62(4):629–41. doi: 10.1093/geront/gnab047 (PMC9019644; doi:10.1093/geront/gnab047)
Supplement: gnab047_suppl_Supplementary_Materials [file gnab047_suppl_supplementary_materials.docx]

**Online Supplementary Material**

Supplementary Table 1. Qualitative questions on meaningful activity in mild dementia in focus groups and individual interviews

| **Qualitative questions for people with dementia** |
| --- |
| - What do you think about the following statement? ‘STAYING ACTIVE’ |
| - Do you think it is important to STAY ACTIVE? |
| - What helps you STAY ACTIVE? |
| - What can get in the way of STAYING ACTIVE? |
| - What activities do you enjoy and are meaningful to you? |
| **Qualitative questions for family carers** |
| - What do you think about the following statement? ‘STAYING ACTIVE in mild dementia’ |
| - Do you think it is important for your relative to STAY ACTIVE? |
| - What helps your relative STAY ACTIVE? |
| - What can get in the way of your relative STAYING ACTIVE? |
| - What activities does your relative enjoy and are meaningful to him/her? |

Supplementary Material A. Search Strategy of the Review of Theoretical Models

1. successful aging/
2. activity theory/
3. resilience/
4. compensation/
5. purposeful activity/
6. meaningful activity/
7. enjoyable activities/
8. well-being/
9. activity engagement/
10. optimisation/
11. 1 or 2 or 3 or 4 or 5 or 6 or 7 or 8 or 9 or 10
12. older people/
13. late-life/
14. dementia/
15. cognitive impairment/cognitive decline
16. 12 or 13 or 14 or 15
17. theory/
18. framework/
19. model/
20. 17 or 18 or 19

Supplementary Table 2. Demographics of people with dementia and family carers taking part in service user consultations (N=36)

| **Variables** | **Mean (SD) or N (%)** |
| --- | --- |
| *People with dementia*  N = 16 |  |
| Age (years) | 81.2 (7.5) |
| Sex  Female | 9 (56) |
| Ethnicity  White British  Irish/Other White  Black Caribbean/Black African  Other | 10 (66)  4 (16)  1 (9)  1 (9) |
| Dementia type  Alzheimer’s Disease  Mixed dementia  Vascular dementia  Frontotemporal dementia | 12 (75)  2 (13)  1 (6)  1 (6) |
| MMSE | 25.7 (3.0) |
|  |  |
| *Carers*  N = 20 |  |
| Age (years) | 63.1 (13.4) |
| Sex  Female | 13 (65) |
| Ethnicity  White British  Irish/Other White  Black Caribbean/Black African  Other | 16 (80)  2 (10)  1 (5)  1 (5) |
| Relationship to participant  Spouse/partner  Child/Child in law  Other | 12 (60)  7 (35)  1 (5) |

Supplementary Table 3. Theoretical models identified as relevant to meaningful activity in mild dementia for developing theory-driven intervention methods

|  | **Theoretical model** | **Brief theoretical explanation** | **Proposed mechanisms** |
| --- | --- | --- | --- |
|  | ***Activity oriented theories*** |  |  |
|  | Selection, optimisation and compensation (Baltes & Baltes, 1990) | Selection (narrowing of goals), optimisation (means to achieve selected goals), and compensation (seeking external resources for support) | Agency, sense of competence, resources |
|  | Successful ageing (Rowe & Kahn 1998) | Successful ageing defined by low-disease related disability, achieving maximum function and active engagement with life | Agency, mastery, interpersonal relations |
|  | Activity theory (Cavan et al., 1949) | Maintaining high activity patterns and values necessary for well-being; life satisfaction depends on how active the individual is | Roles, continuity, self-esteem |
|  | ***Psycho-social theories*** |  |  |
|  | Continuity theory of ageing (Atchley 1989) | Continuity of social behavior, accomplishing objectives tied to past experiences, and need to maintain internal and external structures | Identity, roles, resources |
|  | Self-determination theory (Deci & Ryan, 1985) | The need to experience competence, meaningful social connections, and autonomy aligned with one’s goals and needs | Competence, agency, roles |
|  | Life span theory of control (Heckhausen & Schulz 1995), self-esteem (Atchley 1991), and life review and identity theory (Erikson’s et al 1986) | Producing behavior-event contingencies via control over the environment, stability of self-esteem as indicator of healthy functioning, identity formation and developing a life history | Feelings of control, positive self-concept, identity |
|  | Social-cognitive theory (Bandura 1986) | Reciprocal nature of human functioning, whereby personal, behavioral and environmental influences interact to produce human agency | Agency, self-efficacy, mastery |
|  | Socio-emotional selectivity theory (Carstensen 1992) | Focus shift towards maximisation of meaningful activities in the present, sources of meaning and meaningful interactions as sources of positive affect | Positive affect, deriving meaning |
|  | Theory of copying and adaptation with active ageing ([Salazar-Barajas](https://www.ncbi.nlm.nih.gov/pubmed/?term=Salazar-Barajas%20ME%5BAuthor%5D&cauthor=true&cauthor_uid=28934037) et al., 2017) | Engaging in adaptive processes to cope with disease, by re-establishing the ‘system’ and meeting core human needs by socially connecting with others | Social support, relationships with others |
|  | Ways of coping model (Tanner 2007) | Adapting to change through maintaining valued activities that support feelings of continuity, personal responsibility and sense of ‘doing well’ | Valued activities, competence, identity, self-esteem |
|  | Hierarchical model of needs in dementia ([Scholzel-Dorenbos](https://www.ncbi.nlm.nih.gov/pubmed/?term=Scholzel-Dorenbos%20CJ%5BAuthor%5D&cauthor=true&cauthor_uid=20155528) et al., 2010) | Identification of fundamental needs which includes maintenance of self-esteem, self-image, belongingness through attachment and activities, safety and physiological needs | Self-esteem, mastery, relationships, safety, physical health |
|  | Stress process model in early dementia ([Hilgeman](https://www.ncbi.nlm.nih.gov/pubmed/?term=Hilgeman%20MM%5BAuthor%5D&cauthor=true&cauthor_uid=19363019) et al., 2014) | People with early dementia engage in meaning-based coping which includes acceptance, continuity, and maintaining social engagement and autonomy | Continuity, deriving meaning |
|  | ***Ecological theories of ageing*** |  |  |
|  | Ecological model of ageing (Lawton, 1980) | Behavior is a function of competence, with ‘place’ key in deriving meaning and agency-driven patters of activity through daily routines | Agency, daily routine, competence |
|  | Ecological framework of place (Moore 2014) | People enact activities motivated by their own abilities, with ‘place’ ruling the pattern of activities, and ‘roles’ co-enacted | Agency, roles, place |
|  | The Preventive Corrective Proactive model (Kahana et al., 2014) | Behavioral efforts to build one’s resources and achieve person-environment fit to support autonomy and prevent problem situations | Agency, autonomy |

Supplementary Table 4. Content of the STAYING ACTIVE intervention as informed by the different stages of development

| **Objectives** | **Description of objectives** | **Evidence** | **Theory/mechanisms** |
| --- | --- | --- | --- |
| **Motivation and awareness of staying active** | - Enjoy staying active - Maintain activity - Accept loss and compensate - Optimize choices - Living environment | Qualitative interviews, expert consultations, and literature | - Selection optimization compensation (SOC) - Successful Aging - Activity theory of aging - Self-determination theory - Ecological theories of aging - Socio-emotional selectivity theory |
| **Behavioral activation** | - Encourage activity - Promote stimulation - Continuity of the self | Qualitative interviews, expert consultations, and literature | - Continuity theory and identity formation, agency |
| **The therapeutic experience** | - Attitude of the therapist - Therapeutic bond - Acceptance of relaxation and physical activity exercises | Qualitative interviews, expert consultations, and literature | - Coping and stress models |
| **Facilitators of staying active** | - Encouragement and positive affect - Problem solving - Mastery and agency | Qualitative interviews, expert consultations, and literature | - Self-esteem and emotional well-being and theory of control |
| **Barriers of staying active** | - Lack of sustainment due to dementia - Physical health barriers - Lack of knowledge about the person and understanding of dementia - Social and environmental barriers | Qualitative interviews, expert consultations, and literature | - Ecological theories of aging, coping and stress models |
| **Coping with loss** | - Adaptation - Compensation - Optimize resources - Negative emotions | Qualitative interviews, expert consultations, and literature | - Ecological theories of ageing - SOC - Coping models |

Supplementary Table 5. Template for Intervention Description and Replication checklist describing STAYING ACTIVE

| **Item** | **Description** |
| --- | --- |
| Name | STAYING ACTIVE (STAYing well and active – schedulINg meaninGful and enjoyAble aCTIvities to promote Vitality and wEll-being) |
| Theory and goal | STAYING ACTIVE is based on behavioral activation principles, and aims to support and encourage people with dementia to engage and/or maintain engagement in meaningful and enjoyable activities |
| Materials | Participant and carer workbook providing information, and specific topics of discussion  Workbook of specific examples of activities  Relaxation and physical activity exercises  Diary  Dyads supported to engage in STAYING ACTIVE using standardized materials |
| Intervention | Intervention is provided by psychology graduates which are trained by the research team using standardized resources |
| Mode of delivery | The intervention is delivered one-to-one to both participant and carer |
| Location | Participants’ home |
| When and how much | Over 8 sessions delivered for 1 hour, once a week, over 8-12 weeks |
| Tailoring | Tailored to each individual, additional home visits and provision of additional resources if necessary |
| Modifications | Depending on person’s age, and overall health |
